# Uncovering the correlation between neurotransmitter-specific functional connectivity and multidimensional anxiety in a non-clinical cohort

**DOI:** 10.1007/s00406-024-01879-9

**Published:** 2024-08-27

**Authors:** C. Saiz-Masvidal, V. De la Peña-Arteaga, S. Bertolín, I. Martínez-Zalacaín, A. Juaneda-Seguí, P. Chavarría-Elizondo, M. Subirà, J. M. Menchón, M. A. Fullana, C. Soriano-Mas

**Affiliations:** 1 Psychiatry and Mental Health Group, Neuroscience Program, Institut d’Investigació Biomèdica de Bellvitge - IDIBELL, L’Hospitalet de Llobregat, Spain; 2 Department of Clinical Sciences, School of Medicine, Universitat de Barcelona - UB, L’Hospitalet de Llobregat, Spain; 3 Sant Pau Mental Health Research Group, Institut de Recerca Sant Pau, Sant Pau – Campus Salut Barcelona, Barcelona, Spain; 4 CIBERSAM, Instituto de Salud Carlos III, Madrid, Spain; 5 Radiology Department, Hospital Universitari de Bellvitge, L’Hospitalet de Llobregat, Carrer de Feixa Llarga SN, Barcelona, 08907 Spain; 6 Mental Health Department, Parc Taulí Hospital Universitari, Neuroscience and Mental Health Research Area, Institut d’Investigació i Innovació Parc Taulí (I3PT), Sabadell, Spain; 7 Institut d’Investigacions Biomèdiques August Pi i Sunyer, Barcelona, Spain; 8 Hospital Clinic, Barcelona, Spain; 9 Department of Social Psychology and Quantitative Psychology, Institute of Neurosciences, University of Barcelona, Barcelona, Spain

**Keywords:** Anxiety, Monoamines, Functional magnetic resonance imaging, Brain connectivity, Non-clinical samples

## Abstract

Research on anxiety faces challenges due to the wide range of symptoms, making it difficult to determine if different aspects of anxiety are linked to distinct neurobiological processes. Both alterations in functional brain connectivity (FC) and monoaminergic neurotransmitter systems are implicated as potential neural bases of anxiety. We aimed to investigate whole-brain FC involving monoaminergic nuclei and its association with anxiety dimensions in 178 non-clinical participants. Nine anxiety-related scales were used, encompassing trait and state anxiety scores, along with measures of cost-probability, hypervigilance, reward-punishment sensitivity, uncertainty, and trait worry. Resting-state functional magnetic resonance imaging data were acquired, focusing on seven brainstem regions representing serotonergic, dopaminergic, and noradrenergic nuclei, with their FC patterns voxel-wise correlated with the scales. All models underwent family-wise-error correction for multiple comparisons. We observed intriguing relationships: trait and state anxiety scores exhibited opposing correlations in FC between the dorsal raphe nucleus and the paracingulate gyrus. Additionally, we identified shared neural correlates, such as a negative correlation between the locus coeruleus and the frontal pole. This connection was significantly associated with scores on measures of probability, hypervigilance, reward sensitivity, and trait worry. These findings underscore the intricate interplay between anxiety dimensions and subcortico-cortical FC patterns, shedding light on the underlying neural mechanisms governing anxiety.

**Supplementary Information:**

The online version contains supplementary material available at 10.1007/s00406-024-01879-9.

## Introduction

Anxiety is a prevalent emotional experience characterized by feelings of tension, worry, and apprehension. It is often triggered by uncertain dangers related to potential adverse future events [1–4]. While anxiety serves as an adaptive response to cope with potential threats, its dysfunctional expression can lead to the development of anxiety disorders [3]. Recent theoretical frameworks, such as the Uncertainty and Anticipation Model of Anxiety (UAMA) [5], propose that anxiety is a multidimensional phenomenon consisting of five distinct dimensions: inflated estimates of threat cost and probability, increased threat attention and hypervigilance, deficient safety learning, avoidant behavior and thoughts, and intolerance of uncertainty. This multidimensional nature presents challenges in anxiety research due to its symptom heterogeneity [6]. Additionally, while most anxiety research has been conducted in clinical samples, the dimensional conceptualization of anxiety suggests the existence of a continuum between non-clinical and clinical anxiety [7]. Studying samples of individuals with subclinical symptoms or without a formal diagnosis of an anxiety disorder provides an opportunity to assess the neurobiological correlates of anxiety without the typical confounding effects found in clinical samples, such as medication and comorbidities. However, it remains uncertain whether non-clinical anxiety represents a unified phenomenon or possesses a multidimensional nature [1].

In this context, a precise description of the underlying neurobiology of anxiety remains elusive and inherently complex [2, 8]. Understanding the neural circuitry of anxiety requires consideration of symptom variability [9] and describing how specific symptoms relate to abnormal brain processes [10]. It has been postulated that dysfunction in specific neurotransmitter systems and their receptors may underlie the heterogeneity of anxiety’s manifestations [4, 11, 12]. Specifically, the neurotransmitter systems involving serotonin (5-HT), dopamine (DA), and norepinephrine (NE) have been implicated in anxiety-like behaviors [13]. While these neurotransmitter systems also play roles in various other functions [14, 15], extensive research has established the anxiolytic properties of drugs targeting these systems [16]. Monoaminergic neurons, located in discrete nuclei within the midbrain and hindbrain, project long axons to innervate the cortex and subcortical structures. These neurotransmitter systems have been implicated in activating widespread neural systems or networks that play an important role in emotions, arousal, attention and other functions such as motivated behaviour, including reward processing, reinforcement learning, and behavioural flexibility [14, 15], which have been linked to different models to understand anxiety. Well-documented anxiolytic properties of drugs that act primarily on monoaminergic systems have implicated 5-HT, NE, and DA in the pathogenesis of anxiety disorders [16]. A large body of research has confirmed the crucial role of the 5-HT neurotransmitter system in the neural processing of anxiety [17–22], and evidence highlights that the serotonin system likely plays various roles in the anxiety-like behavioral regulation [23]. Different models of anxiety linked to a dual action of serotonin on anxiety have been proposed, those dependent on response suppression, such as the punishment response, which involve potential or distant threats and are based on an amygdala-dependent threat assessment process, and other models of anxiety dependent on response production, which involve immediate threats and are based on the dorsal periaqueductal gray induced flight and fight response. The serotonergic system projects from the two upper raphe nuclei; the dorsal raphe nucleus (DRN) and nucleus centralis superior (NCS) to the forebrain [13]. Although the role of the NCS in the regulation of anxiety has received less attention than that of the DRN, there is substantial evidence to support its involvement [24]. Likewise, there is evidence for the involvement of DA in anxiety modulation in different parts of the brain, including the amygdala and hippocampus [4, 22, 25], playing a significant role in motivation and reward-motivated behaviour [26]. This neurotransmitter is synthesized in dopaminergic neurons located in the midbrain substantia nigra pars compacta (SNc) and the ventral tegmental area (VTA) [27]. There is also evidence of a relationship between noradrenergic brain system and anxiety-associated behaviours [28, 29]. Most NE neurons are located in the locus coeruleus (pons), with projections throughout the cerebral cortex and multiple subcortical areas, including hippocampus, amygdala, thalamus, and hypothalamus. This neuroanatomical formation of the noradrenergic system makes it well suited to modulate brain function rapidly and globally in response to changes in the environment, as occurs during the presentation of stressors [28]. This study sought to examine the neural basis of multidimensional anxiety in a non-clinical population with a focus on neurotransmitter systems. We aimed to uncover variations and commonalities in neural substrates associated with different dimensions of anxiety by analyzing whole-brain functional connectivity patterns related to three major neurotransmitter systems. Our findings have the potential to guide the development of targeted and effective treatments and preventative strategies for addressing various aspects of anxiety in both clinical and non-clinical populations.

## Materials and methods

### Participants

A total of 178 healthy participants (92 females, 51.7%; mean age ± SD = 25.51 ± 4.71 years) were evaluated in this study.

Participants were enrolled as part of a prospective longitudinal investigation into the behavioral and neural predictors of anxiety. To ensure their eligibility for the study, all potential participants underwent a comprehensive screening process conducted through a secure web system. This screening process included the completion of demographic questionnaires, a review of medical history, and a standardized mental health assessment. Subsequently, a telephone interview was conducted by a qualified medical doctor (MD) who administered the Mini International Neuropsychiatric Interview (MINI) [30] to confirm that potential participants met the inclusion/exclusion criteria.

Inclusion criteria mandated that participants be between the ages of 18 and 36 and express a willingness to undergo neuroimaging assessments. Exclusion criteria encompassed individuals with a current or previous severe medical disorder (as reported by the participants), current or past mental disorders (with the exception of current anxiety disorders), or those currently engaged in substance abuse (excluding occasional use of alcohol, other recreational drugs, or tobacco).

Upon meeting the specified inclusion and exclusion criteria, eligible participants provided written informed consent before participating in any study-related procedures. Ethical approval for this study was granted by the Institutional Review Board at Bellvitge Hospital.

All participants underwent a resting-state functional magnetic resonance imaging (fMRI) session at Bellvitge University Hospital (see below).

### Assessment of anxiety

To comprehensively evaluate anxiety measures for each participant, a battery of well-established anxiety questionnaires was administered. These included scores for state and trait general anxiety, as well as a worry score. We also aimed to assess the different dimensions of the UAMA model [5]. However, since there is no established self-report method for assessing deficient safety learning, this measurement was not included. The State-Trait Anxiety Inventory (STAI), developed by Spielberger et al. [31], is a widely recognized instrument for assessing anxiety. It consists of two subscales: the STAI-T measures trait anxiety, reflecting a general predisposition to worry, especially in situations that threaten self-esteem, while the STAI-S assesses state anxiety, capturing momentary feelings of apprehension, tension, nervousness, and worry. Both subscales cover behavioral, cognitive, emotional, and physiological aspects. The STAI is valued for its sensitivity as a predictor of anxiety disorders [5].

The Outcome Probability Questionnaire (OPQ) and the Outcome Cost Questionnaire (OCQ) [32], are complementary tools designed to assess individuals’ perceptions of risk and the emotional cost of negative outcomes. It is a 24 items scale, evenly split to cover scenarios of physical threat (12 items) and social threat (12 items). In the OPQ, participants are asked to rate the likelihood of experiencing various negative outcomes within the next year, providing a quantitative measure of perceived risk. Conversely, the OCQ asks participants to evaluate how distressing or severe these outcomes would be should they actually occur, aiming to gauge the emotional impact or cost associated with these negative expectations. These tools therefore offer insights into two critical dimensions of anxiety and risk perception: the probability and the emotional consequences of adverse events.

The Brief Hypervigilance Scale (HYPV), derived from the original Hypervigilance Scale developed by Bernstein et al. [33], is a streamlined assessment tool consisting of five items specifically designed to measure intense fear responses indicative of hypervigilance. This concise instrument adeptly captures the essence of the original scale, focusing on how individuals perceive and react to their environment under conditions of perceived threat. Hypervigilance is a critical component in the psychopathology of anxiety disorders, where it contributes significantly to the heightened sensitivity to potential dangers. This heightened state of alertness often leads individuals to misinterpret ambiguous situations as threatening and to exaggerate the significance of minor or non-threatening events [34].

The Sensitivity to Punishment and Sensitivity to Reward Questionnaire (SPSRQ) [35] is a tool designed to measure individual differences in behavioral responses driven by two core neuropsychological systems: the Behavioral Inhibition System (BIS) and the Behavioral Activation System (BAS). This 24-item scale is divided into two primary subscales. The Sensitivity to Punishment (SPSRQ-SP) subscale evaluates how individuals respond to potential aversive outcomes. It assesses the degree of behavioral inhibition an individual exhibits when faced with scenarios that might lead to punishment, failure, or other negative consequences. This subscale also explores worry and cognitive processes that are activated by the threat of adverse results, reflecting a person’s sensitivity to potential losses or punishments. The Sensitivity to Reward (SPSRQ-SR) subscale is focused on the propensity to seek out and respond to rewarding situations. It describes the behavioral activation that occurs when individuals are presented with opportunities to gain positive outcomes or pleasures through their actions. This aspect of the questionnaire assesses how likely individuals are to engage in behavior aimed at achieving rewards, highlighting differences in how people are motivated by potential gains.

The Intolerance of Uncertainty Scale (IUS) [36], is a psychological assessment tool designed to measure how individuals respond to uncertain or ambiguous situations. Consisting of 27 items, the IUS evaluates a range of emotional, cognitive, and behavioral reactions that individuals exhibit when faced with uncertainty. The scale explores how discomfort with the unknown impacts an individual’s emotions, leading to anxiety or distress. It also assesses cognitive responses, which include a tendency to ruminate on possible futures or the inability to process ambiguous information without distress. Behaviorally, the IUS measures the extent to which individuals attempt to control future outcomes, reflecting actions taken to minimize uncertainty or avoid uncertain situations altogether. These behaviors often manifest as an increased need for predictability, over-preparation for potential scenarios, or avoidance of situations where outcomes are not guaranteed.

The Penn State Worry Questionnaire (PSWQ) [37], is a specialized assessment tool developed to quantify the trait of worry among adults. This 16-item scale is designed to capture the nuances of chronic worry as a distinct psychological trait. It evaluates several key dimensions of worry: its generality, excessiveness, and uncontrollability. Generality refers to the wide range of topics about which an individual tends to worry, from personal health to social interactions to professional obligations, indicating how pervasive worry is across various life domains. Excessiveness measures the degree to which these worries are perceived as more intense or more frequent than what might be considered typical or reasonable. Uncontrollability assesses an individual’s ability (or inability) to suppress worrisome thoughts, reflecting how much control one feels over their worry process. All 178 participants completed the STAI tests, while 160 participants successfully completed the entire battery of anxiety questionnaires.

### fMRI data acquisition and pre-processing

MRI data were acquired using a 3.0 Tesla MRI scanner (Ingenia, Philips Medical Systems, Eindhoven, Best, Netherlands) equipped with a 32-channel phased-array head coil. For the resting-state fMRI sequences, a total of 240 single-shot gradient-echo echo-planar imaging (EPI) volumes were obtained. The acquisition parameters included a repetition time of 2,000 ms, echo time of 25 ms, and a pulse angle of 90º. The field of view was set at 240 mm, and the images had an 80 × 80 pixel matrix, resulting in voxel sizes of 3 × 3 × 3 mm with no gap. Each whole-brain volume comprised 40 interleaved slices, aligned parallel to the anterior-posterior commissure line. The entire resting sequence had a duration of 8 min.

To facilitate the registration of EPI data into standard MNI space and to extract the overall individual gray matter volume, a high-resolution T1-weighted anatomical scan was also acquired to facilitate the registration of EPI data into standard MNI space and for extracting individual global grey matter volume. Specifically, the parameters were as follows: a three-dimensional fast-spoiled gradient, inversion-recovery sequence with 220 contiguous slices (repetition time, 10.5 ± msec; echo time, 4.8 msec; flip angle, 8°) in a 24-cm field of view, with a 320 × 320 pixel matrix and isotropic voxel sizes of 0.75 × 0.75 × 0.75 mm.

Functional neuroimaging data were processed and analyzed on a Microsoft Windows platform using MATLAB 9.3 (Release 2017b, The MathWorks, Inc.) and the CONN Functional Connectivity toolbox (Functional Connectivity SPM Toolbox v20.b; www.nitrc.org/projects/conn). The default Montreal Neurological Institute (MNI) preprocessing pipeline implemented in the CONN toolbox was utilized for functional image preprocessing. Specifically, all images underwent the standard pre-processing steps of: i./ realignment and slice-timing correction, to account for head motion and slice-timing differences; ii/. normalization, structural volumes were segmented and normalized to the MNI space to define gray/white matter and cerebrospinal fluid segments, and this normalization was then applied to the functional data; and iii/. smoothing, data were smoothed with an 8-mm full-width at half maximum (FWHM) isotropic Gaussian kernel to increase signal-to-noise ratio and to account for anatomical variability across participants. In addition, blood oxygenation level–dependent noise from white matter and cerebrospinal fluid was characterized with the principal component–based aCompCor method and, in a subsequent denoising step, regressed out from blood oxygenation level–dependent signal time series. A linear detrending term was also used to remove linear/quadratic/cubic trends. Images were despiked prior to regression by applying a squashing function to reduce the influence of potential outlier scans, and subjects with more than 33% of invalid scans, 70 out of 240, were excluded from the study. Finally, band-pass filtering was performed with a frequency window between 0.008 and 0.09 Hz. Importantly, to ensure image quality, the sequences were inspected for artifacts before and after each step. Fifteen individuals, from an original sample of 193 participants, were excluded because of image artifacts, excessive movement, or gross abnormalities upon visual inspection of the images.

### Definition of regions of interest (ROI)

To assess the functional connectivity of brainstem nuclei, ROIs were defined using the MarsBar ROI toolbox (http://marsbar-toolbox.github.io/marsbar). The ROIs were created as 3-mm radial spheres [38] centered on specific bilateral MNI coordinates. The following brainstem nuclei were targeted for investigation:

Serotonergic System: DRN: MNI coordinates [x = 0, y=-26, z=-18], and NCS: MNI coordinates [x = 0, y=-32, z=-24] [39].

Dopaminergic System: VTA: MNI coordinates [x = 0, y=-15, z=-12] [40]; SNc: Bilateral ROIs with MNI coordinates [x = ± 7, y=-18, z=-17] [41].

Noradrenergic System: LC: Bilateral ROIs with MNI coordinates [x = ± 4, y=-34, z=-32] [42].

To prevent potential signal overlap between seeds caused by spatial smoothing, we ensured that the seeds within each hemisphere were separated by more than 8 mm (equivalent to 1 FWHM). This separation was determined using the equation:

$$\:\sqrt{{\left(x1-x2\right)}^{2}+{\left(y1-y2\right)}^{2}+{\left(z1-z2\right)}^{2}}$$

**Fig. 1:**
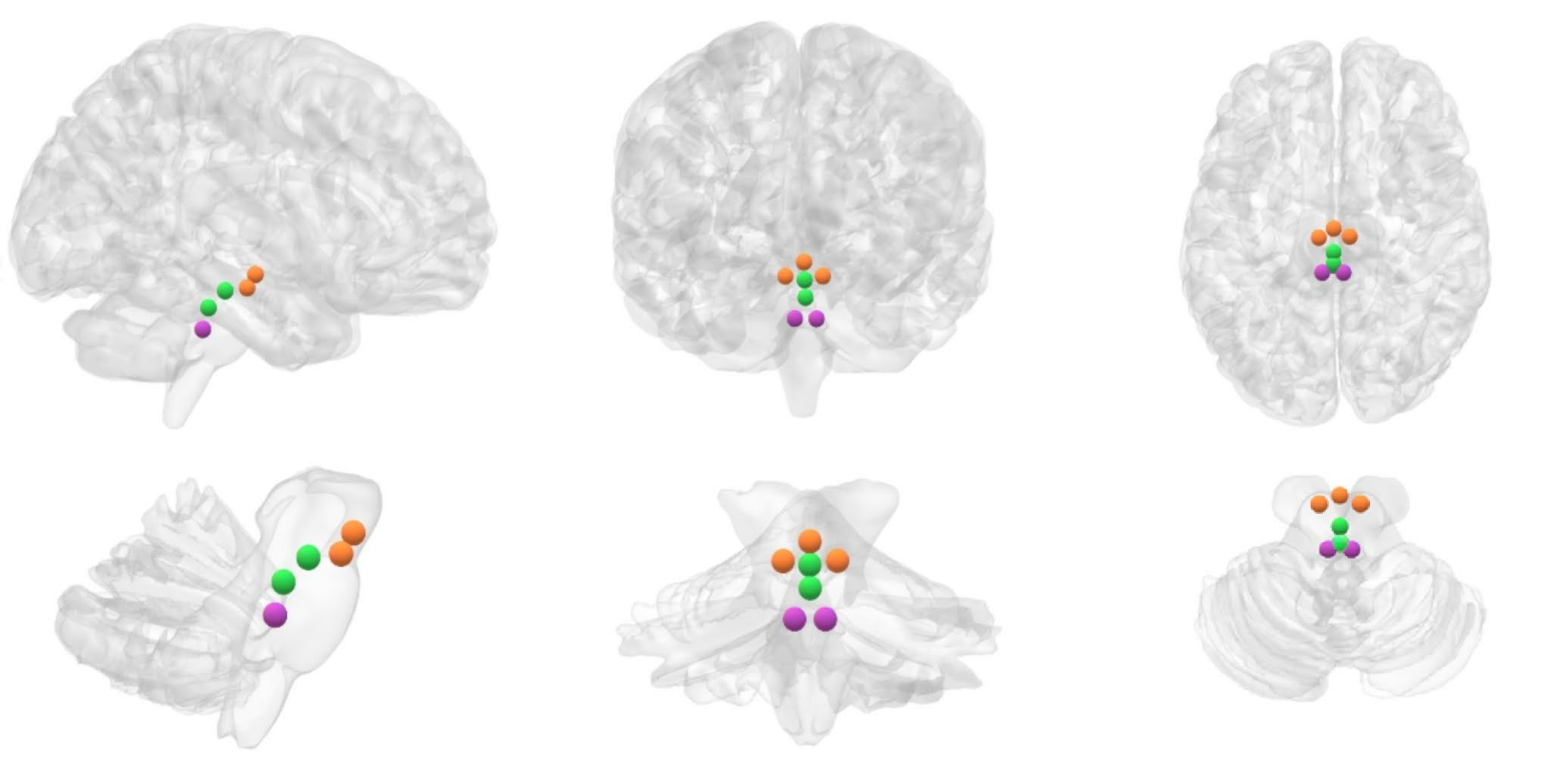
The figure displays three-dimensional brain renderings showcasing sagittal (left), coronal (center), and axial (right) views of both the entire brain (top) and the brainstem (bottom). These renderings feature 3-mm radial spheres, color-coded in green, orange, and purple, which functioned as seed regions of interest for investigating specific neurotransmitter-related nuclei in this study. The green seeds correspond to the serotonergic system, with the DRN located in a more rostro-ventral position, and the NCS situated just caudal to it. Orange seeds represent the dopaminergic system, featuring the centrally positioned VTA and the laterally separated SNc in a more caudal location. Lastly, the purple seeds represent the noradrenergic system, corresponding to the bilateral LC

We employed normalized-space masks, which were applied to individually normalized images of all participants. This approach enabled us to calculate the average blood-oxygen-level-dependent (BOLD) signal time series within the defined ROIs. This procedure facilitated the analysis of functional connectivity patterns involving brainstem nuclei and their interactions with other brain regions. The use of a central coordinate for defining the ROI upon which the analysis sphere is based minimizes the inclusion of signals from bordering areas, thus reducing the risk of signal contamination from nearby regions [13, 38, 43–45]; Fig. 1 provides a visual representation of the ROIs used to investigate the functional connectivity of the brainstem systems under study.

### Image analyses

We conducted data analyses using the CONN toolbox. In the first-level analyses, individual seed-based correlation maps were computed to assess functional connectivity during resting state between each of the seven seed ROIs and the entire brain.

To explore the relationship between functional connectivity and anxiety measures, we performed second-level multivariate general linear model analyses, modeling data across multiple subjects. Independent regression analyses were carried out for each ROI’s functional connectivity map in relation to various anxiety scores (see Table 1). For general anxiety measures corresponding to the STAI, the regression model included both trait and state anxiety scores, with each controlling for the other in their respective cases. This approach enabled a more specific examination of the distinct contributions of each STAI measure to functional connectivity.

We assessed both positive and negative correlations, with a significance threshold set at *p* < 0.05, cluster-level family-wise-error (FWE) corrected. Cluster-level FWE correction is employed to adjust the significance level accounting for the multiple comparisons made in voxel-wise analyses. This method specifically identifies the minimum number of contiguous voxels that must exceed a predetermined intensity threshold (i.e., *p* < 0.001) for a cluster to be considered statistically significant.

## Results

### Sample description

Anxiety levels, as measured by STAI scores, were evaluated across the entire sample comprising 178 individuals. Within this group, we further examined specific anxiety dimensions in the subset of 160 participants, who completed the entire battery of anxiety questionnaires, although only 157 individuals completed the PSWQ assessment. Table 1 presents comprehensive descriptive statistics for the study samples, including the different anxiety scores, while Supplementary Table S1 details the correlations among these scores.

**Table 1:** Sociodemographic and clinical characteristics of the sample

Assessment	Statistics			
	Mean ± SD	Min.	Max.	Normative Values (mean ± SD)^1^
		**General anxiety assessment** (*n* = 178)		
**Age**, yr	25.51 *±* 4.71	19	36	
**Gender**, female (n; %)	92; 51.7%			
**STAI-T**	18.73 *±* 9.10	1	44	18,96 *±* 10.0
**STAI-S**	10.82 *±* 6.18	0	32	15,87 *±* 9.92
		**Dimensional anxiety assessment** (*n* = 160)		
**Age**, yr	25.08 *±* 4.64	19	36	
**Gender**, female (n; %)	80; 50%			
**OPQ**	38.59 *±* 20.46	0	100	N.A.
**OCQ**	93.86 *±* 35.72	4	162	N.A.
**HYPV**	3.06 *±* 2.59	0	12	N.A.
**SPSRQ-SP**	7.64 *±* 4.60	0	20	11.65 *±* 5.27
**SPSRQ-SR**	9.19 *±* 4.51	1	23	12.18 *±* 4.48
**IUS**	50.49 *±* 15.44	27	105	53.81 *±* 17.88
**PSWQ** ^2^	25.49 *±* 9.47	11	48	31.0 *±* 10.1

HYPV = Brief Hypervigilance Scale; IUS= Intolerance of Uncertainty Scale; Max = maximum; Min = minimum; N.A.: Not available for the Spanish reference population; OCQ = Probability and Costs Questionnaire – Cost ; OPQ = Probability and Costs Questionnaire – Probability; PSWQ = Penn State Worry Questionnaire; SD = standard deviation; SPSRQ-SP = Sensitivity to Punishment and Sensitivity to Reward Questionnaire – Sensitivity to Punishment ; SPSRQ-SR= Sensitivity to Punishment and Sensitivity to Reward Questionnaire – Sensitivity to Reward; STAI-S = *State Anxiety Inventory* ; STAI-T = *Trait Anxiety Inventory*

^1^Normative values derived from [46] (STAI), [35] (SPSRQ), [47] IUS, and [48] PSWQ

^2^*n*=157

### Association between general anxiety scores and neurotransmitter-specific patterns of functional connectivity

To investigate the neural underpinnings of global anxiety, we conducted an extensive study that involved analyzing the relationship between STAI Trait (STAI-T) and State (STAI-S) scores and functional connectivity seed maps across the entire brain.

**Fig. 2:**
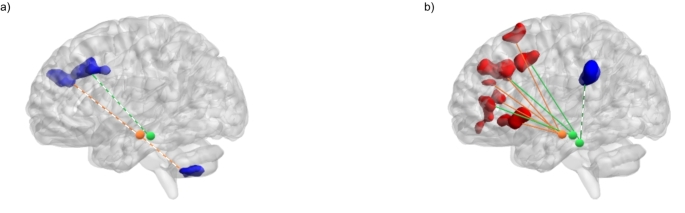
Brain regions exhibiting significant correlations with anxiety scores are as follows: (**a**) We observed negative correlations between the STAI-T scores and whole-brain functional connectivity with the DRN, illustrated by green connections, as well as the bilateral SNc, represented by orange connections. These correlations encompassed the paracingulate gyrus and the right frontal pole and cerebellum. (**b**) For STAI-S scores, we found positive correlations with whole-brain functional connectivity with the DRN (rostral green seed) and the right SNc (orange). These positive correlations extended to various brain regions, including the paracingulate gyrus, the left superior, middle, and orbital frontal regions, and the right superior temporal gyrus. Additionally, we observed a negative correlation between STAI-S scores and whole-brain functional connectivity with the NCS (caudal green seed), which involved the left supramarginal gyrus. Positive correlations are indicated by solid lines, and negative correlations are indicated by dashed lines

We found that STAI-T scores exhibited negative correlations with connectivity between the DRN and the paracingulate gyrus, as well as between the SNc and the frontal pole and cerebellum (refer to Fig. 2a).

In contrast, STAI-S scores displayed positive correlations with the functional connectivity between the DRN and the paracingulate and cingulate gyri. Additionally, STAI-S scores showed a positive correlation with functional connectivities primarily originating from the right SNc, with the NCS contributing to a lesser extent, and a widespread pattern of frontal regions, including the superior and middle frontal gyri and the orbital cortex. Furthermore, we identified a negative correlation between NCS and the supramarginal gyrus connectivity (Fig. 2b). These results are reported in Table 2 and supplementary Figure S1.

**Table 2:** Whole-brain correlations between functional connectivity patterns and STAI scores (controlling for the complementary STAI score)

Brainstem seed	MNI coordinates			k	*p*_FWE−corr_	t-statistic	Pearson correlation coefficient (*r*)	Anatomic location
	x	y	z					
						**STAI-T**		
					*Negative correlations*			
DRN (5HT)	+ 04	+ 22	+ 38	455	0.001	4.54	**-0.32**	**R/L Paracingulate Gyrus (and Cingulate Gyrus)**
L SNc (DA)	+ 34	+ 38	+ 28	273	0.009	4.82	**-0.34**	**R Frontal Pole**
R SNc (DA)	-02	-60	-46	341	0.002	4.89	**-0.34**	**R/L Cerebellum 8 9**
						**STAI-S**		
					*Positive correlations*			
DRN (5HT)	+ 04	+ 32	+ 32	399	0.002	5.03	**0.35**	**R/L Paracingulate Gyrus**
	-04	+ 40	+ 08	314	0.004	4.28	**0.31**	**Cingulate Gyrus**,** anterior division**
NCS (5HT)	-22	+ 30	+ 38	201	0.048	3.91	**0.28**	**L Superior Frontal Gyrus**
R SNc (DA)	-56	+ 14	00	339	0.002	4.41	**0.32**	**L Frontal Orbital Cortex**
	-32	+ 10	+ 40	299	0.010	5.08	**0.36**	**L Middle Frontal gyrus**
	+ 10	+ 30	+ 12	255	0.014	5.32	**0.37**	**Cingulate Gyrus**,** anterior division**
	-02	+ 16	+ 64	244	0.013	4.81	**0.34**	**L Superior Frontal Gyrus**
	+ 02	+ 44	+ 36	183	0.043	4.30	**0.31**	**R Superior Temporal Gyrus (and Paracingulate Gyrus)**
					*Negative correlations*			
NCS (5HT)	-58	-42	+ 34	215	0.037	4.25	**-0.31**	**L Supramarginal Gyrus**,** posterior division**

DA = dopamine; *k* = cluster extent; L = left; LC = locus coeruleus; NCS = nucleus centralis superior; NE = norepinephrine; R = right; SFG = superior frontal gyrus; SNc = substantia nigra pars compacta; 5-HT = serotonin

### Association between dimension-specific anxiety scores and neurotransmitter-specific patterns of functional connectivity

Here, our objective was to investigate the neural correlates of various anxiety dimensions by analyzing questionnaire responses, specifically OPQ and OCQ, HYPV, SPSRQ-SP and -SR, IUS, and PSWQ, in conjunction with functional connectivity maps of brainstem nuclei at a whole-brain level.

**Fig. 3:**
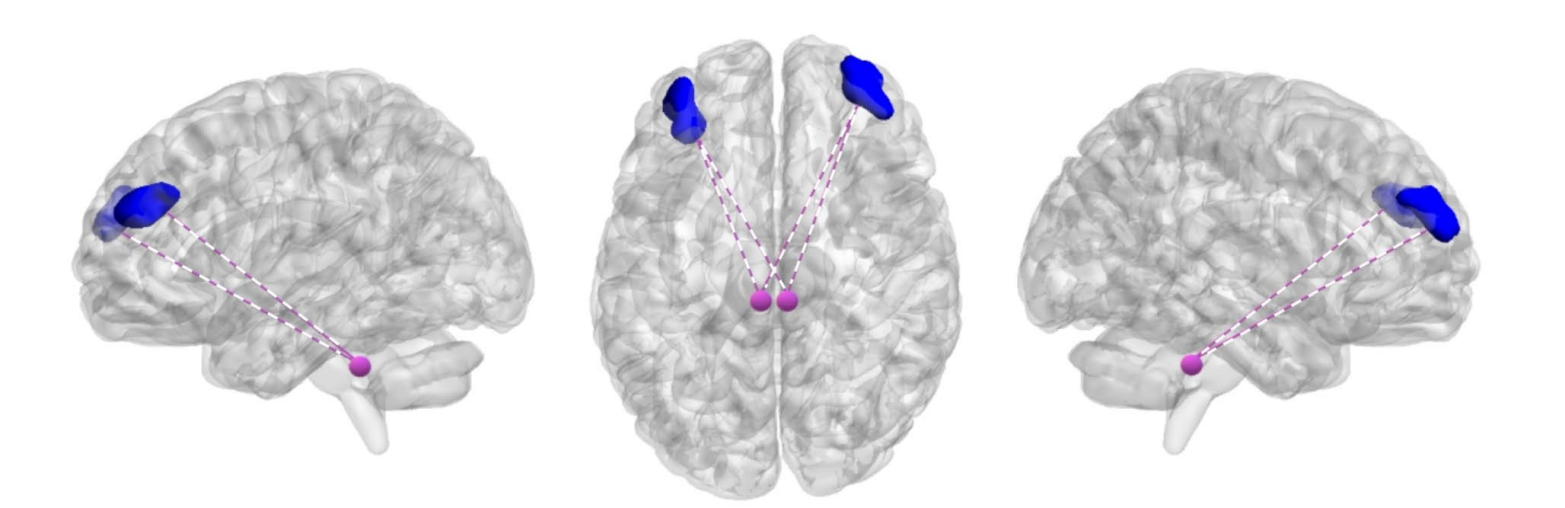
The figure demonstrates a consistent negative correlation between bilateral frontal pole regions and the LC across associations with OPQ, HYPV, SPSRQ-SP, and PSWQ scores

Our findings unveiled significant correlations for OPQ, revealing a positive association between the DRN and bilateral precentral and postcentral gyrus. Additionally, the right SNc exhibited positive correlations with the right superior and left inferior temporal gyrus (both anterior and posterior divisions). Conversely, OPQ displayed a negative correlation with functional connectivity between the bilateral LC and the left frontal pole (Fig. 3). Conversely, OCQ showed negative correlations between the DRN and the right insula, as well as between the left SNc and the brainstem pons.

**Fig. 4:**
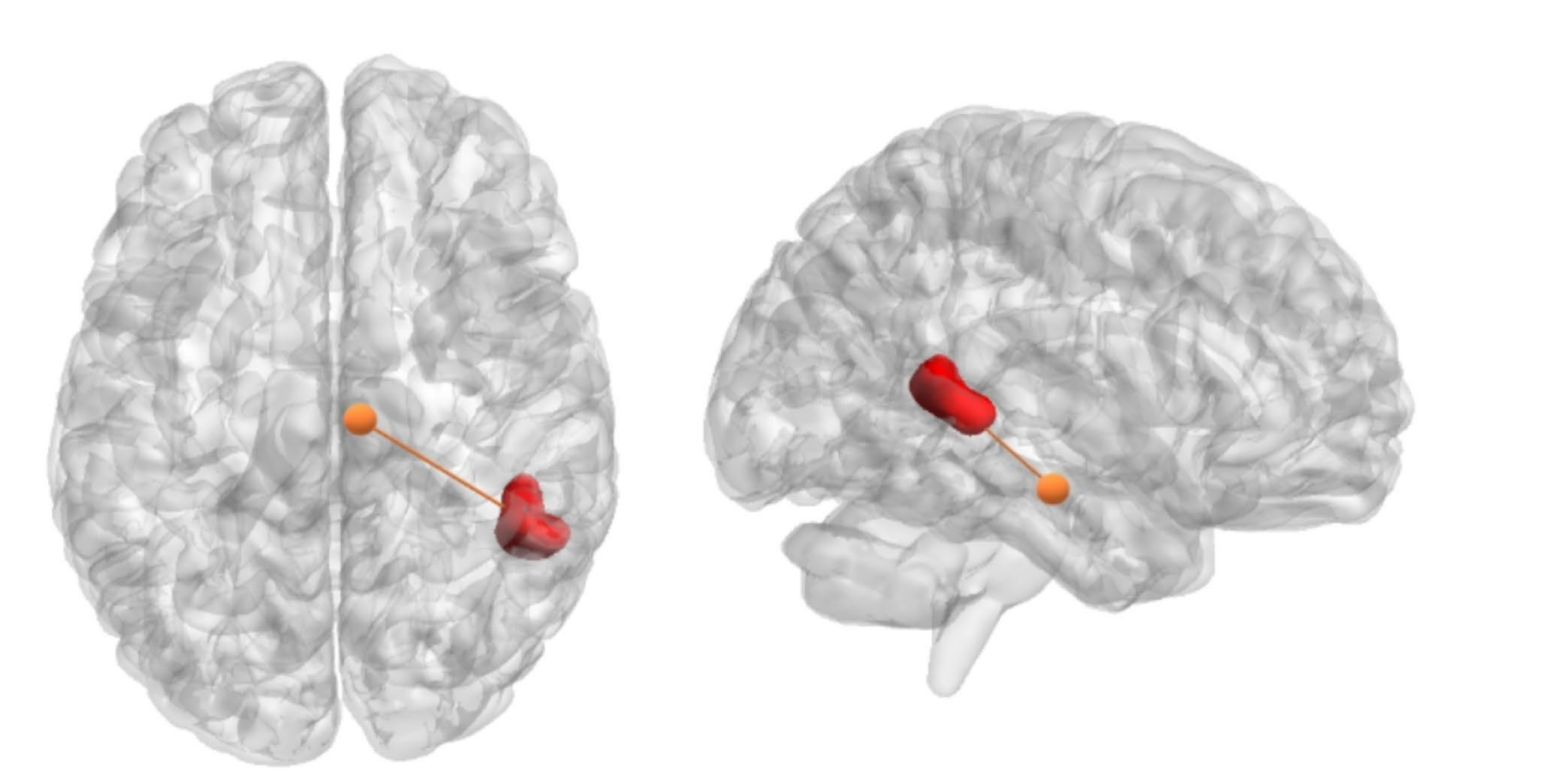
The figure portrays the middle temporal gyrus, particularly its temporooccipital segment, revealing a positive correlation with the right SNc in relation to both HYPV and SPSRQ-SP scores

Regarding HYPV scores, we observed positive correlations between the DRN and the middle temporal gyrus (posterior division), the right SNc and cerebellum crus I-II, and the middle temporal gyrus (temporooccipital part). Furthermore, the right LC displayed a positive correlation with the left lingual gyrus, while bilateral LC exhibited a negative correlation with bilateral frontal pole (see Figs. 3 and 4).

Our analysis of SPSRQ-SP revealed a positive correlation between the right SNc and the right middle temporal gyrus (temporooccipital part and posterior division). Conversely, the left SNc showed negative correlations with the orbital frontal cortex (including the insula), and the right LC exhibited a negative correlation with the frontal pole (see Figs. 3 and 4). In the case of SPSRQ-SR, the right LC exhibited both positive correlations with the left hippocampus and bilateral thalamus, and negative correlations with the medial frontal cortex.

Finally, for PSWQ scores, we identified a negative correlation between bilateral LC and the left frontal pole (see Fig. 3). It’s worth noting that no statistically significant results were observed for IUS in this study. These results are summarized in Table 3 and supplementary Figures S2 and S3.

**Table 3:** Whole-brain correlations between functional connectivity patterns and dimensional anxiety scores

Brainstem seed	MNI coordinates			k	*p*_FWE−corr_	t-statistic	Pearson correlation coefficient (*r*)	Anatomic location
	x	y	z					
						**OPQ**		
					*Positive correlations*			
DRN (5-HT)	42	-16	36	549	0.000	4.55	**0.34**	**R Precentral and postcentral gyrus**
	-42	-20	36	430	0.001	5.50	**0.40**	**L Precentral and postcentral gyrus**
R SNc (DA)	58	-10	-04	241	0.012	3.99	**0.30**	**R Superior Temporal gyrus**,** ant. division**
	-62	-40	-22	172	0.050	4.24	**0.32**	**L Inferior Temporal gyrus**,** post. division**
					*Negative correlations*			
R LC (NE)	-46	44	18	225	0.016	4.24	**-0.32**	L Frontal Pole * ^**1**^
L LC (NE)	-46	42	18	179	0.041	4.55	**-0.34**	L Frontal Pole * ^**1**^
						**OCQ**		
					*Negative correlations*			
DRN (5-HT)	30	-22	20	246	0.015	4.36	**-0.33**	**R Insular cortex**
L SNc (DA)	08	-30	-42	301	0.004	4.64	**-0.35**	**Brainstem (pons)**
						**HYPV**		
					*Positive correlations*			
DRN (5-HT)	70	-24	-06	428	0.001	4.37	**0.33**	**R Middle Temporal gyrus**,** post. division**
R SNc (DA)	32	-60	-42	229	0.015	4.04	**0.31**	**R Cerebellum Crus1 / Cerebellum Crus2**
	50	-30	02	226	0.016	4.08	**0.31**	**R Middle Temporal gyrus**, temporooccipital part *^2^
R LC (NE)	-20	-46	-08	195	0.030	4.42	**0.33**	**L Lingual gyrus**
					*Negative correlations*			
R LC (NE)	-34	56	26	255	0.009	3.96	**-0.30**	L Frontal Pole * ^**1**^
	-28	24	14	224	0.016	4.65	**-0.35**	**L Paracingulate gyrus**
	38	60	18	203	0.025	3.87	**-0.29**	R Frontal Pole * ^**1**^
L LC (NE)	36	58	18	343	0.002	4.78	**-0.36**	R Frontal Pole * ^**1**^
	-36	46	22	291	0.004	4.13	**-0.31**	L Frontal Pole * ^**1**^
						**SPSRQ-SP**		
					*Positive correlations*			
R SNc (DA)	48	-46	06	267	0.007	4.09	**0.31**	**R Middle Temporal gyrus**, temporooccipital part *^2^
	56	-14	-10	218	0.019	5.14	**0.38**	**R Middle Temporal gyrus**,** post. division**
					*Negative correlations*			
L SNc (DA)	-30	28	-02	248	0.012	5.19	**-0.38**	**L Frontal Orbital cortex / Insular cortex**
R LC (NE)	-32	56	26	242	0.011	4.06	**-0.31**	L Frontal Pole * ^**1**^
						**SPSRQ-SR**		
					*Positive correlations*			
R LC (NE)	-26	-28	-06	209	0.022	4.53	**0.34**	**L Hippocampus**
	-04	-16	10	204	0.015	4.36	**0.33**	**Thalamus**
					*Negative correlations*			
R LC (NE)	-06	54	-16	331	0.002	4.19	**-0.32**	**Frontal Medial cortex**
						**PSWQ**		
					*Negative correlations*			
R LC (NE)	-34	42	32	483	0.001	4.38	**-0.33**	L Frontal Pole * ^**1**^
L LC (NE)	-34	44	24	244	0.024	4.45	**-0.34**	L Frontal Pole * ^**1**^

DA = dopamine; DRN = dorsal raphe nucleus; *k* = cluster size; L = left; LC = locus coeruleus; NCS = nucleus centralis superior; NE = norepinephrine; R = right; SNc = substantia nigra pars compacta; 5-HT = serotonin; *^1^displayed in Fig. 3; *^2^displayed in Fig. 4

In the table presented above, the results exhibiting consistency across various questionnaires are denoted by an asterisk (*). These common findings are further illustrated in the accompanying figures below.

## Discussion

This study represents a pioneering endeavor as the first to evaluate functional connectivity from neurotransmitter nuclei seeds in assessing the multidimensionality of anxiety symptomatology. In addition to assessing the FC of monoaminergic-system correlates of general trait and state anxiety measurements, our neuroimaging data revealed distinctive functional brain patterns for different anxiety dimensions, underscoring their neurobiological diversity. These findings confirm that each dimension reflects unique aspects of anxiety, constituting independent psychological processes. Interestingly, we also observed common neural features across some evaluated questionnaires, suggesting potential cross-cutting neural functional underpinnings among certain anxiety-related symptoms.

In our investigation of general anxiety, we observed distinct patterns between trait and state anxiety, a finding consistent with previous research by Saviola et al. [3]. Analyzing the STAI-T questionnaire, we observed a negative correlation between 5-HT nuclei and the paracingulate gyrus, extending to the cingulate gyrus. Notably, anxiety patients [48, 49] have shown decreased connectivity in this region, which is associated with fear-related modality-general activation patterns [50]. Moreover, the paracingulate gyrus has been linked to theory of mind and self-monitoring functions, such as visual self-recognition, autobiographical memory, conflict monitoring, verbal self-monitoring, and self-generated thoughts [51]. These components are believed to play a role in initiating and maintaining anxiety symptoms [52]. Conversely, when assessing the STAI-S, we observed a positive correlation in the same region with 5-HT nuclei. Previous research indicated that the paracingulate cortex is active at rest, suggesting it might represent a “default” mode of introspective functioning [53]. We speculate that individuals with trait anxiousness and hypervigilance might have an underactivated paracingulate gyrus, while those experiencing acute anxiety, may have an overactivated paracingulate gyrus.

We also observed a negative correlation between STAI-T scores and the connectivity of DA nuclei with both the cerebellum and the right frontal pole. The involvement of the cerebellum in emotional processing, aversive learning, memory, and social cognition has been well-established in prior research [54–56]. Notably, the cerebellum’s influence on higher cognitive processes extends to individual differences in anxiety vulnerability [57]. Moreover, the cerebellum exhibits strong connectivity with various areas known to be part of the anxiety circuitry, including those associated with neurotransmitters involved in fear and anxiety [58]. Additionally, it plays a role in the anticipation and prediction processes that accompany fear and anxiety-related conditions [59]. As for the right frontal pole, it is critical for the effective regulation of emotions, including controlling negative emotions like anxiety [16, 20]. Specifically, the frontal pole/middle frontal gyrus (MFG) is believed to provide critical “top-down” governance [60], a directional influence from higher cortical control areas (especially the PFC) to subcortical areas [61], and has been linked to impaired decision-making for complex or difficult choices, possibly reflecting increased impulsivity or reduced cognitive processing [62]. Furthermore, this region is proposed to interrupt ongoing endogenous attentional processes and reorient attention to an exogenous stimulus [63]. Consistent with our results, Zhao et al. [64] postulated that decreased activity in the MFG is indicative of the alterations involved in the neural basis of anxiety. They demonstrated decreased amplitude of low frequency fluctuation values in the MFG in patients with an anxiety disorder compared to non-anxious participants, along with lower activity in bilateral anterior lateral prefrontal cortices, which aligns with our region-result and its involvement in the regulation of emotional processing.

In clear contrast to the above findings, STAI-S scores showed positive correlations with connectivity between several left prefrontal regions and the 5-HT and DA nuclei, including the superior frontal gyrus and the orbitofrontal cortex (OFC), known for its hyperactivity in the presence of anxiety-laden cognitions [65]. Moreover, the OFC has been linked to interpreting ambiguous stimuli as threatening [5]. These findings demonstrate that while trait and state anxiety can be associated with similar brainstem-prefrontal circuits, their underlying neural mechanisms appear to be distinct. Finally, connectivity between the supramarginal gyrus and 5-HT nuclei was negatively correlated with STAI-S scores, a result that concurs with previous studies relating this region with anxiety [66] and the decrease in functional connectivity of this region in anxious individuals [67].

On the other hand, in the analyses of the neural correlates of the anxiety subdimensions, we observed that some of them share the same negative correlation with the frontal pole/MFG connectivity with bilateral NE nuclei. These subdimensions include OPQ, HYPV, SPSRQ-SP, and PSWQ. Hence, it appears that receiving lower NE input in this prefrontal brain region cuts across different dimensions of anxiety. In combination with the above results for general anxiety measurements, these findings suggest that hypoactivation of the frontal pole/MFG might represent one of the underlying neural bases of trait anxiety, which is consistent across several of its subdimensions. In contrast, hyperactivation, along with a broader prefrontal area, might be associated with acute anxiety experienced at a specific moment.

Among the shared neural patterns across anxiety subdimensions, the OCQand SPSRQ-SP exhibited negative correlations with the 5-HT and DA nuclei, respectively, and the insular cortex. This limbic cortex region has been previously associated with anxiety and risk sensitivity [68–70], influencing emotional states and subjective estimates of potential future threats [5]. Additionally, the medial temporal gyrus (MTG) displayed a positive correlation with the DA nucleus for HYPV and SPSRQ-SP. The MTG is integral to cognitive processing of emotions and emotion regulation [71] and has been linked to anxiety symptoms in non-clinical populations [72, 73].

Furthermore, the OPQ showed a positive correlation between the 5-HT nucleus and the bilateral precentral and postcentral gyrus, regions associated with increased risk perception [68] and anxiety symptoms [74]. Additionally, the DA nucleus displayed positive correlations with the right superior temporal gyrus and inferior temporal gyrus, both involved in social cognition and risk perception [64, 68, 75]. For HYPV, the right NE nucleus exhibited positive correlations with the lingual gyrus and cerebellum Crus I and II, regions contributing to social mentalizing and optimal predictions about social interactions [56, 76]. SPSRQ-SP showed a negative correlation with the DA nucleus and a cluster involving the insula and OFC, both implicated in threat processing and avoidant behaviors [5]. Lastly, SPSRQ-SR displayed negative correlations with the frontal medial cortex and positive correlations with the left hippocampus and thalamus, regions associated with reward evaluation and modulation of responses to reward-related cues [77–81].

Our work aligns with the Research Domain Criteria (RDoC) initiative, emphasizing a dimensional approach to understanding mental disorders and investigating underlying neural circuits for different domains of function [82, 83]. While previous studies have mainly focused on clinical samples, this research takes a multidimensional perspective, shedding light on the neural bases of non-clinical anxiety. The findings supported the hypothesis that each dimension of anxiety exhibits specificity in its neurological substrates, although some common regions are also present. Notably, all three neurotransmitter systems studied (5-HT, DA, and NE) were implicated in the neural correlates of multidimensional anxiety. However, the locus coeruleus-NE system emerged as the most implicated nucleus, playing a central role in fear and anxiety models and modulating arousal states and adaptive behavior [84–86]. Dysregulation of the NE system may contribute to the pathogenesis of anxiety dimensions [87].

Understanding the neurobiological mechanisms underlying anxiety-related processes and evaluating neurotransmitter-related brain functionality can offer a novel theoretical framework for anxiety. This multidimensional perspective allows for a comprehensive consideration of the entire spectrum of anxiety symptoms based on their neural correlates, linking functional connectivity to the neurochemical framework. Moreover, this research has potential implications for subclinical treatment by identifying vulnerable individuals at risk and offering specific preventive measures tailored to affected dimensions. A transdiagnostic approach addressing altered dimensions across various anxiety disorders can enhance the clinical community’s ability to improve therapeutic efficacy. Future research into the neurobiology of anxiety-related subdimensions and their neurochemical basis is crucial for translating these findings into promising clinical applications.

The study’s limitations warrant consideration. Firstly, the use of seed-based neuroimaging techniques to assess functional connectivity may not provide a direct and accurate representation of how neurotransmitter nuclei mediate hypo- or hyperactivity in functionally connected brain regions. As a result, drawing definitive conclusions from the results becomes challenging. However, we chose this approach due to its theoretical and innovative nature, offering a novel direction in understanding the neural basis of multidimensional anxiety. Nonetheless, further research is necessary to validate and interpret the results, given the significant practical implications of linking functional neuroimaging to the neurochemical framework. Another limitation stems from the lack of a standardized theoretical framework that would classify specific cognitive dimensions constituting anxiety and their respective assessment methods. Additionally, the Hypervigilance questionnaire’s content validity may be questioned due to its limited number of items. While we believe that the questionnaires employed in this study adequately encompass the dimensional representation of the anxiety spectrum, it is essential to move towards standardization to characterize and assess anxiety dimensions in a more valid and consistent manner. Addressing these limitations in future research would enhance the robustness and validity of our findings and contribute to a more comprehensive understanding of anxiety’s multidimensional nature.

## Conclusions

In conclusion, this investigation highlights the notion that each anxiety dimension represents an independent psychobiological process, characterized by its distinct neural functional underpinnings. However, there are also shared neural bases underlying certain anxiety dimensions, as evidenced by the decreased norepinephrine-mediated functional connectivity in the frontal pole, suggesting some common neural-based psychological characteristics among them. Additionally, our findings reaffirm the involvement of monoaminergic systems in the etiology of anxiety, shedding light on the neurobiological underpinnings of this complex condition. By examining the neural correlates of anxiety from a multidimensional perspective, this research provides valuable insights into the neural mechanisms driving anxiety-related processes in non-clinical populations. These findings have the potential to contribute significantly to the development of preventive measures, offering tailored approaches to address various anxiety dimensions and enhance overall mental well-being. By advancing our understanding of anxiety’s multifaceted nature, we can make significant strides in the field of mental health research and improve strategies for promoting emotional resilience and effective coping in diverse populations.

## Electronic supplementary material

Below is the link to the electronic supplementary material.

Supplementary Material 1
