# Supplementary material for: Uncovering the correlation between neurotransmitter-specific functional connectivity and multidimensional anxiety in a non-clinical cohort
Source: Eur Arch Psychiatry Clin Neurosci. 2024 Aug 27;276(4):1911–25. doi: 10.1007/s00406-024-01879-9 (PMC13233946; doi:10.1007/s00406-024-01879-9)
Supplement: Supplementary file 1 — Supplementary Material 1 [file 406_2024_1879_MOESM1_ESM.docx]

**Supplementary material**

**Table S1**. Detailed correlations among different anxiety dimensions

|  | | STAI-T | STAI-S | OPQ | OCQ | HYPV | SPSRQ-SP | SPSRQ-SR | IUS |
| --- | --- | --- | --- | --- | --- | --- | --- | --- | --- |
| STAI-S | Pearson correlation  Sig. (bilateral) | 0.431  0.000 |  |  |  |  |  |  |  |
| OPQ | Pearson correlation  Sig. (bilateral) | 0.366  0.000 | 0.360  0.000 |  |  |  |  |  |  |
| OCQ | Pearson correlation  Sig. (bilateral) | 0.196  0.013 | 0.266  0.001 | 0.348  0.000 |  |  |  |  |  |
| HYPV | Pearson correlation  Sig. (bilateral) | 0.359  0.000 | 0.477  0.000 | 0.325  0.000 | 0.179  0.024 |  |  |  |  |
| SPSRQ-SP | Pearson correlation  Sig. (bilateral) | 0.529  0.000 | 0.431  0.000 | 0.493  0.000 | 0.370  0.000 | 0.451  0.000 |  |  |  |
| SPSRQ-SR | Pearson correlation  Sig. (bilateral) | 0.172  0.030 | 0.44  0.585 | 0.295  0.000 | 0.116  0.145 | 0.301  0.000 | 0.212  0.007 |  |  |
| IUS | Pearson correlation  Sig. (bilateral) | 0.504  0.000 | 0.427  0.000 | 0.459  0.000 | 0.345  0.000 | 0.462  0.000 | 0.594  0.000 | 0.296  0.000 |  |
| PSWQ | Pearson correlation  Sig. (bilateral) | 0.601  0.000 | 0.498  0.000 | 0.406  0.000 | 0.274  0.001 | 0.527  0.000 | 0.594  0.000 | 0.152  0.057 | 0.652  0.000 |


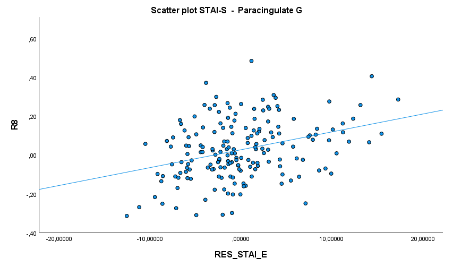
**Figure S1.** Scatter plots of representative results in Fig. 2 of the manuscript showing positive correlations between bilateral paracingulate gyrus and DRN and between superior frontal gyrus and NCS and right SNc associated with STAI-S covaried by the effect of the STAI-T; and negative correlations between DRN and bilateral paracingulate gyrus and between left SNc and right frontal pole associated with STAI-T covaried by the effect of the STAI-S.

a) STAI-S: positive correlation


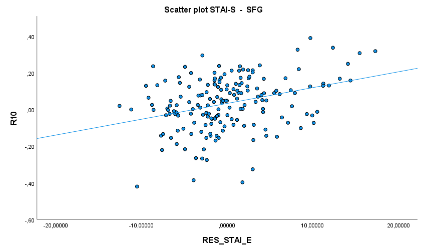
b) STAI-S: positive correlation

| Brainstem seed | MNI coordinates | | | *k* | *p*_FWE-corr_ | t-statistic | Pearson correlation coefficient (r) | Anatomic location |
| --- | --- | --- | --- | --- | --- | --- | --- | --- |
|  | x | y | z |  |  |  |  |  |
| DRN (5HT) | +04 | +32 | +32 | 399 | 0.002 | 5.03 | 0.35 | Paracingulate Gyrus |

| Brainstem seed | MNI coordinates | | | *k* | *p*_FWE-corr_ | t-statistic | Pearson correlation coefficient (r) | Anatomic location |
| --- | --- | --- | --- | --- | --- | --- | --- | --- |
|  | x | y | z |  |  |  |  |  |
| NCS (5HT) | -22 | +30 | +38 | 201 | 0.048 | 3.91 | 0.28 | L Superior Frontal Gyrus |

| Brainstem seed | MNI coordinates | | | *k* | *p*_FWE-corr_ | t-statistic | Pearson correlation coefficient (r) | Anatomic location |
| --- | --- | --- | --- | --- | --- | --- | --- | --- |
|  | x | y | z |  |  |  |  |  |
| R SNc (DA) | -02 | +16 | +64 | 244 | 0.013 | 4.81 | 0.34 | L Superior Frontal Gyrus |


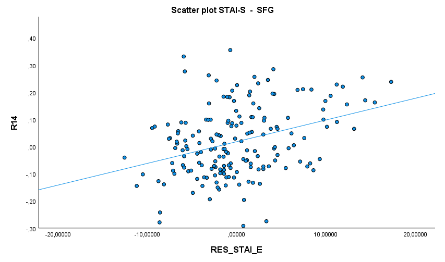
c) STAI-S: positive correlation


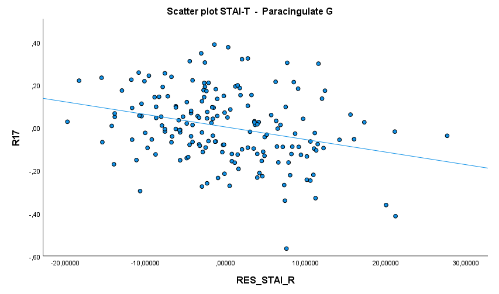
d) STAI-T: negative correlation

| Brainstem seed | MNI coordinates | | | *k* | *p*_FWE-corr_ | t-statistic | Pearson correlation coefficient (r) | Anatomic location |
| --- | --- | --- | --- | --- | --- | --- | --- | --- |
|  | x | y | z |  |  |  |  |  |
| DRN (5HT) | +04 | +22 | +38 | 455 | 0.001 | 4.54 | -0.32 | Paracingulate Gyrus |


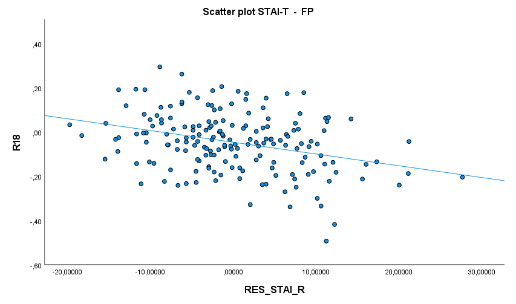


e) STAI-T: negative correlation

| Brainstem seed | MNI coordinates | | | *k* | *p*_FWE-corr_ | t-statistic | Pearson correlation coefficient (r) | Anatomic location |
| --- | --- | --- | --- | --- | --- | --- | --- | --- |
|  | x | y | z |  |  |  |  |  |
| L SNc (DA) | +34 | +38 | +28 | 273 | 0.009 | 4.82 | -0.34 | R Frontal Pole |

**Figure S2.** Scatter plots of the results in Fig. 3 of the manuscript showing the consistency of the negative correlation between bilateral frontal pole regions and LC across associations with OPQ, HYPV, SPSRQ-SP, and PSWQ scores.


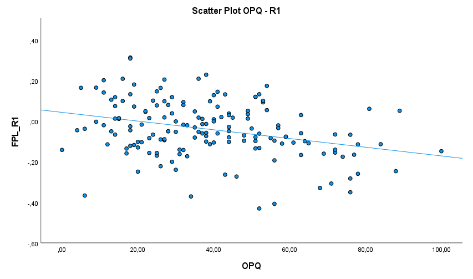


1. OPQ: negative correlation


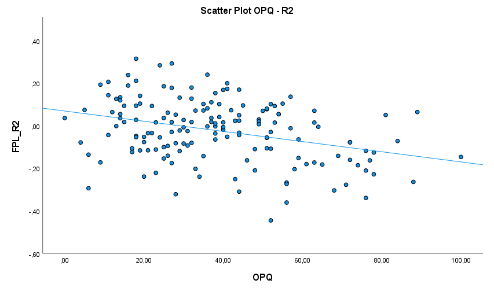


1. OPQ: negative correlations

| Brainstem seed | MNI coordinates | | | *k* | *p*_FWE-corr_ | t-statistic | Pearson correlation coefficient (r) | Anatomic location |
| --- | --- | --- | --- | --- | --- | --- | --- | --- |
|  | x | y | z |  |  |  |  |  |
| R LC (NE) | -46 | 44 | 18 | 225 | 0.016 | 4.24 | -0.32 | L Frontal Pole *^1^ |

| Brainstem seed | MNI coordinates | | | *k* | *p*_FWE-corr_ | t-statistic | Pearson correlation coefficient (r) | Anatomic location |
| --- | --- | --- | --- | --- | --- | --- | --- | --- |
|  | x | y | z |  |  |  |  |  |
| R LC (NE) | -46 | 42 | 18 | 179 | 0.041 | 4.55 | -0.34 | L Frontal Pole *^1^ |

1.
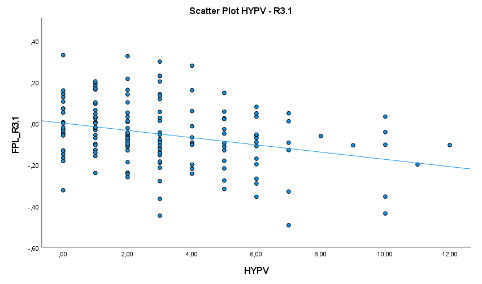
HYPV: negative correlation


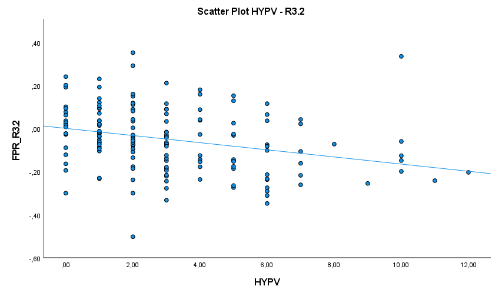


d) HYPV: negative correlation

| Brainstem seed | MNI coordinates | | | *k* | *p*_FWE-corr_ | t-statistic | Pearson correlation coefficient (r) | Anatomic location |
| --- | --- | --- | --- | --- | --- | --- | --- | --- |
|  | x | y | z |  |  |  |  |  |
| R LC (NE) | -34 | 56 | 26 | 255 | 0.009 | 3.96 | -0.30 | L Frontal Pole *^1^ |

| Brainstem seed | MNI coordinates | | | *k* | *p*_FWE-corr_ | t-statistic | Pearson correlation coefficient (r) | Anatomic location |
| --- | --- | --- | --- | --- | --- | --- | --- | --- |
|  | x | y | z |  |  |  |  |  |
| R LC (NE) | 38 | 60 | 18 | 203 | 0.025 | 3.87 | -0.29 | R Frontal Pole *^1^ |


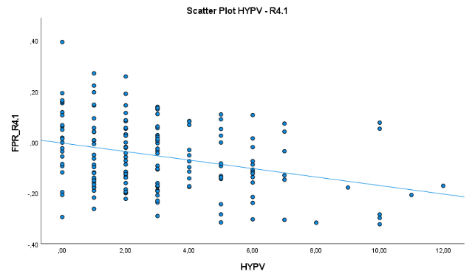
e) HYPV: negative correlation


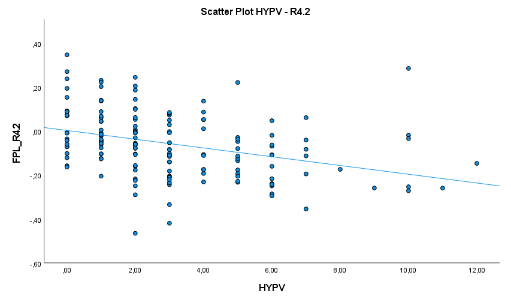


f) HYPV: negative correlation

| Brainstem seed | MNI coordinates | | | *k* | *p*_FWE-corr_ | t-statistic | Pearson correlation coefficient (r) | Anatomic location |
| --- | --- | --- | --- | --- | --- | --- | --- | --- |
|  | x | y | z |  |  |  |  |  |
| L LC (NE) | 36 | 58 | 18 | 343 | 0.002 | 4.78 | -0.36 | R Frontal Pole *^1^ |

| Brainstem seed | MNI coordinates | | | *k* | *p*_FWE-corr_ | t-statistic | Pearson correlation coefficient (r) | Anatomic location |
| --- | --- | --- | --- | --- | --- | --- | --- | --- |
|  | x | y | z |  |  |  |  |  |
| L LC (NE) | -36 | 46 | 22 | 291 | 0.004 | 4.13 | -0.31 | L Frontal Pole *^1^ |


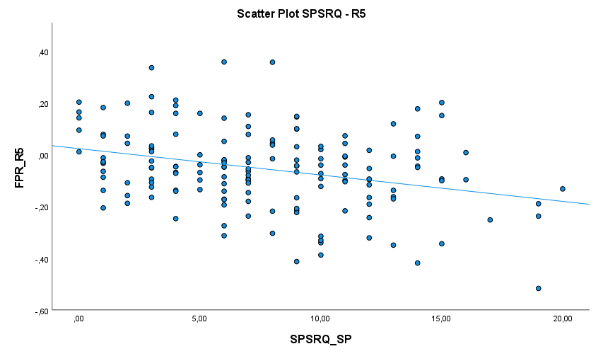


g) SPSRQ-SP: negative correlation

| Brainstem seed | MNI coordinates | | | *k* | *p*_FWE-corr_ | t-statistic | Pearson correlation coefficient (r) | Anatomic location |
| --- | --- | --- | --- | --- | --- | --- | --- | --- |
|  | x | y | z |  |  |  |  |  |
| R LC (NE) | -32 | 56 | 26 | 242 | 0.011 | 4.06 | -0.31 | L Frontal Pole *^1^ |

**
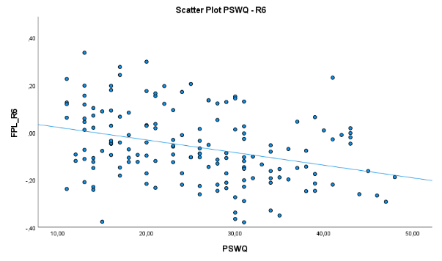
**h) PSWQ: negative correlation

**
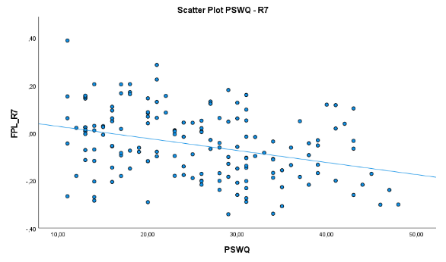
**

i) PSWQ: negative correlation

| Brainstem seed | MNI coordinates | | | *k* | *p*_FWE-corr_ | t-statistic | Pearson correlation coefficient (r) | Anatomic location |
| --- | --- | --- | --- | --- | --- | --- | --- | --- |
|  | x | y | z |  |  |  |  |  |
| R LC (NE) | -34 | 42 | 32 | 483 | 0.001 | 4.38 | -0.33 | L Frontal Pole *^1^ |

| Brainstem seed | MNI coordinates | | | *k* | *p*_FWE-corr_ | t-statistic | Pearson correlation coefficient (r) | Anatomic location |
| --- | --- | --- | --- | --- | --- | --- | --- | --- |
|  | x | y | z |  |  |  |  |  |
| L LC (NE) | -34 | 44 | 24 | 244 | 0.024 | 4.45 | -0.34 | L Frontal Pole *^1^ |

**Figure S3.** Scatter plots of the results in Fig. 4 of the manuscript showing the consistency of the positive correlation between the middle temporal gyrus, particularly its temporooccipital segment, and the right SNc across associations with HYPV and SPSRQ-SP scores.


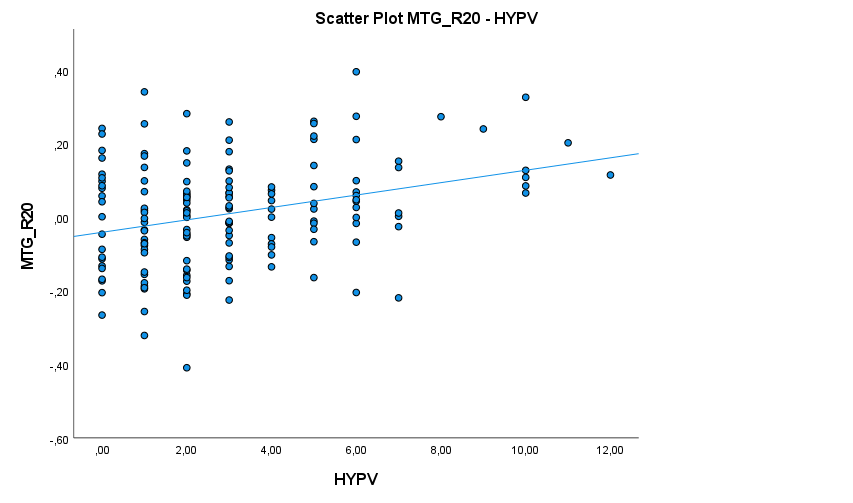


a) HYPV: positive correlation

| Brainstem seed | MNI coordinates | | | *k* | *p*_FWE-corr_ | t-statistic | Pearson correlation coefficient (r) | Anatomic location |
| --- | --- | --- | --- | --- | --- | --- | --- | --- |
|  | x | y | z |  |  |  |  |  |
| R SNc (DA) | 50 | -30 | 02 | 226 | 0.016 | 4.08 | 0.31 | R Middle Temporal gyrus, temporooccipital part *^2^ |


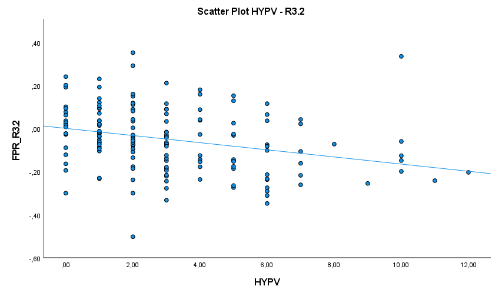

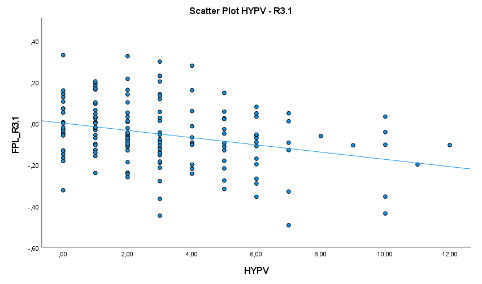


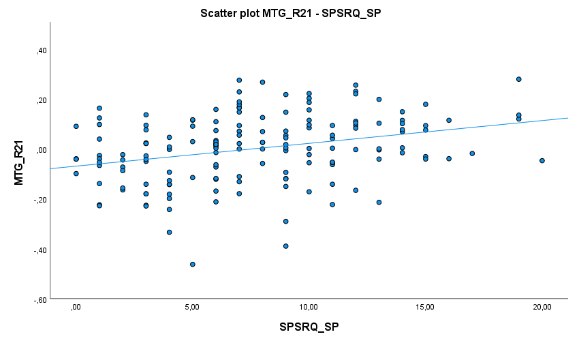


b) SPSRQ-SP: positive correlation

| Brainstem seed | MNI coordinates | | | *k* | *p*_FWE-corr_ | t-statistic | Pearson correlation coefficient (r) | Anatomic location |
| --- | --- | --- | --- | --- | --- | --- | --- | --- |
|  | x | y | z |  |  |  |  |  |
| R SNc (DA) | 48 | -46 | 06 | 267 | 0.007 | 4.09 | 0.31 | R Middle Temporal gyrus, temporooccipital part *^2^ |
